# Executive functioning in preschoolers with 22q11.2 deletion syndrome and the impact of congenital heart defects

**DOI:** 10.1186/s11689-023-09484-y

**Published:** 2023-05-13

**Authors:** Emma Everaert, Jacob A. S. Vorstman, Iris S. Selten, Martijn G. Slieker, Frank Wijnen, Tessel D. Boerma, Michiel L. Houben

**Affiliations:** 1 Institute for Language Sciences, Utrecht University, Trans 10, 3512 JK Utrecht, The Netherlands; 2 Department of Pediatrics, Wilhelmina Children’s Hospital, University Medical Center Utrecht, Lundlaan 6, 3584 EA Utrecht, The Netherlands; 3 Program in Genetics and Genome Biology, Research Institute, and Department of Psychiatry, Hospital for Sick Children, 555 University Avenue, Toronto, M5G 1X8 Canada; 4 Department of Psychiatry, University of Toronto, 250 College Street, Toronto, ON M5T 1R8 Canada; 5 Department of Pediatric Cardiology, Wilhelmina Children’s Hospital, University Medical Center Utrecht, PO Box 85090, 3508 AB Utrecht, The Netherlands

**Keywords:** *22q11.2* deletion syndrome, 22q11DS, Executive functioning, Congenital heart defect, Selective attention, Working memory, Velocardiofacial syndrome, DiGeorge syndrome

## Abstract

**Background:**

Executive functioning (EF) is an umbrella term for various cognitive functions that play a role in monitoring and planning to effectuate goal-directed behavior. The 22q11.2 deletion syndrome (22q11DS), the most common microdeletion syndrome, is associated with a multitude of both somatic and cognitive symptoms, including EF impairments in school-age and adolescence. However, results vary across different EF domains and studies with preschool children are scarce. As EF is critically associated with later psychopathology and adaptive functioning, our first aim was to study EF in preschool children with 22q11DS. Our second aim was to explore the effect of a congenital heart defects (CHD) on EF abilities, as CHD are common in 22q11DS and have been implicated in EF impairment in individuals with CHD without a syndromic origin.

**Methods:**

All children with 22q11DS (*n* = 44) and typically developing (TD) children (*n* = 81) were 3.0 to 6.5 years old and participated in a larger prospective study. We administered tasks measuring visual selective attention, visual working memory, and a task gauging broad EF abilities. The presence of CHD was determined by a pediatric cardiologist based on medical records.

**Results:**

Analyses showed that children with 22q11DS were outperformed by TD peers on the selective attention task and the working memory task. As many children were unable to complete the broad EF task, we did not run statistical analyses, but provide a qualitative description of the results. There were no differences in EF abilities between children with 22q11DS with and without CHDs.

**Conclusion:**

To our knowledge, this is the first study measuring EF in a relatively large sample of young children with 22q11DS. Our results show that EF impairments are already present in early childhood in children with 22q11DS. In line with previous studies with older children with 22q11DS, CHDs do not appear to have an effect on EF performance. These findings might have important implications for early intervention and support the improvement of prognostic accuracy.

**Supplementary Information:**

The online version contains supplementary material available at 10.1186/s11689-023-09484-y.

## Background

The 22q11.2 deletion syndrome (22q11DS; OMIM #192430, #188400, #611867), previously also referred to as DiGeorge or Velo-Cardio-Facial Syndrome, is the most common chromosomal microdeletion syndrome in humans and has an estimated incidence of 1 per 2148 [1]. It results from a hemizygous microdeletion on the long arm of chromosome 22 [2–4]. The syndrome has a widely variable phenotype and symptoms can include, but are not limited to, congenital heart defects (CHD), palatal abnormalities, immunodeficiency, endocrine abnormalities, intellectual disability, and an increased risk for psychiatric disorders [5]. In addition, impairments have been reported in various cognitive domains, including executive functioning (EF) [6].

A recent systematic review of EF abilities in children and adolescents with 22q11DS showed a relative paucity of research on the EF abilities of preschool-aged children with 22q11DS [7]. As EF is related to functional outcomes later in life (see “Clinical importance of EF” section), an accurate description of early EF abilities in children with 22q11DS can have important clinical implications for prognosis and early intervention. Here, we compare EF performance of 3.0- to 6.5-year-old children with 22q11DS to typically developing (TD) peers. Furthermore, we investigate whether the presence of CHD is associated with EF skills in children with 22q11DS, as CHD are common in the 22q11DS population and are associated with EF deficits in the general population [8, 9].

### The organization and development of executive functioning

EF refers to higher-level cognitive functions that regulate lower-level cognitive processes to achieve goal-directed behavior [10–14]. The most commonly proposed EF components are *updating*, *inhibition*, and *shifting* [15]. In early childhood, these components are undifferentiated [16–19]; subsequent differentiation is gradual with distinct developmental trajectories [20–22]. This is in line with the structural and functional development of the prefrontal cortex [23–25], which is the primary brain region associated with EF [26].

**Fig. 1:**
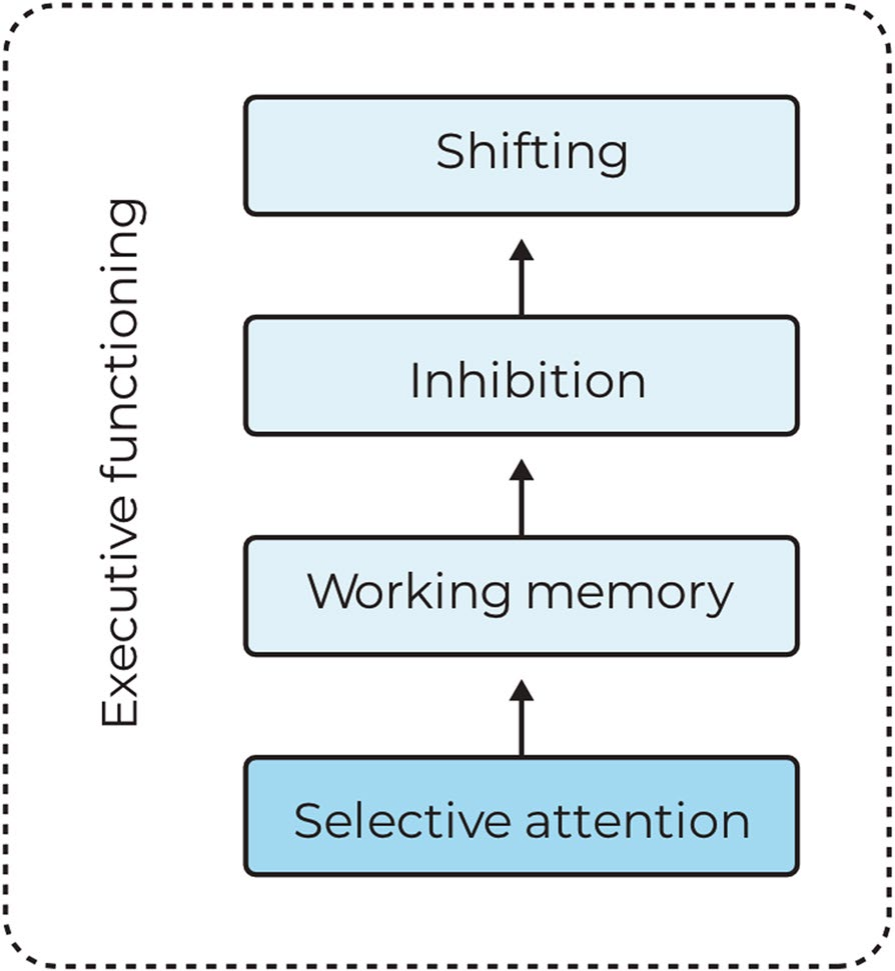
Simplified schematic illustration of EF structure according to the models of [15, 27]

Expanding on the model of Miyake et al. [15], Garon et al. [27] proposed a hierarchical view of EF with selective attention as a basic cognitive function essential for the development of EF (see Fig. 1). Selective attention refers to the ability to direct attentional resources to a specific target, highlighting its features while diminishing target-irrelevant features [28]. Attentional processes rapidly develop during early childhood, with selective attention emerging from 9 months onwards [29]. Indeed, measures of attention during infancy predict EF abilities in toddlerhood [30, 31]. At the age of 2.5 years, selective attention, specifically, has been shown to predict working memory (WM) and inhibition skills at 3 years of age [32]. Thus, given its importance for the development of other EF components, selective attention can be considered a highly relevant function in describing children’s EF profile at the preschool age.

### EF in 22q11DS

A recent systematic review reported impairments in the subdomains of inhibition and shifting in school-aged children and adolescents with 22q11DS [7]. Findings for WM, however, were inconclusive. For verbal WM, the mixed outcomes may be explained by developmental changes. Studies with younger children with 22q11DS have not found differences in verbal WM skills in comparison to TD peers [33, 34], whereas studies with older children have [35, 36]. Verbal WM may thus be a relative strength in early childhood. Several studies on visuospatial WM report weaker performance of children with 22q11DS [35, 37–40], although others observed no difference with TD peers [36, 41–43]. However, most studies report age ranges that span more than 7 years and cover late childhood to adolescence (≥ 8 to ≤ 18 years old), making it difficult to determine whether visuospatial WM is already impaired in early childhood (≤ 7 years old).

Attentional deficits have also been reported in individuals with 22q11DS (e.g., [42, 44–47]). However, selective attention specifically has not yet been studied in detail. One study examined selective attention as a predictor of social cognition and reported that children with 22q11DS (5–13 years) made more errors than TD controls on the selective attention task [48]. To our knowledge, however, there are no studies that have investigated selective attention as a primary outcome in children with 22q11DS.

### Clinical importance of EF

EF has been implicated in many domains of functioning, as well as quality of life, and mental and physical health [12]. For example, EF skills are known to predict later academic achievement and language outcomes for both TD children [49–51] and children with 22q11DS ([35, 52], but see [53]). Moreover, in the general population, EF is associated with later physical and mental health outcomes [54, 55]. In 22q11DS, EF has been shown to relate to adaptive functioning and daily living skills [35, 56]. Accordingly, in the general population, EF impairments have been associated with increased levels of psychopathology [57] and developmental disorders, such as attention deficit hyperactivity disorder and autism spectrum disorder [58–60], all of which occur at increased rates in children with 22q11DS [5, 35, 37, 61, 62]. Deficits in EF have furthermore been suggested to precede the onset of schizophrenia [63–67]. As 22q11DS is the strongest single genetic variant associated with schizophrenia [5, 68], an accurate description of early EF abilities in children with 22q11DS can have important clinical implications for prognosis and early intervention (e.g., [69, 70]).

### Congenital heart defects

**Fig. 2:**
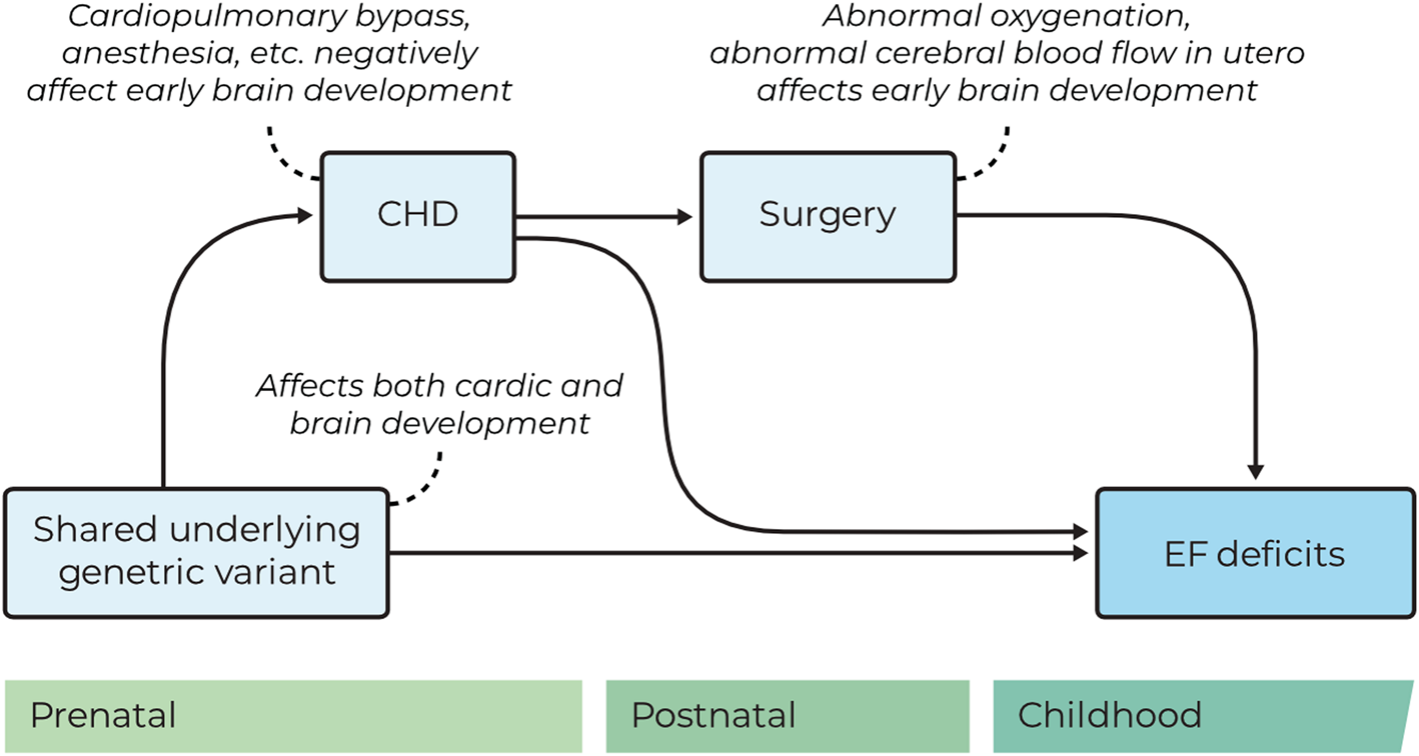
Schematic illustration of the potential causal pathways between CHD and EF deficits

In the general population, the presence of CHDs is associated with poorer EF outcomes [8, 9]. CHDs are common in 22q11DS, with prevalence rates estimated from 31% to as high as 75% [5, 71–74]. Types of CHDs in 22q11DS mostly consist of conotruncal abnormalities and atrioventricular septal defects, including tetralogy of Fallot, ventricular septal defects, interrupted aortic arch, and truncus arteriosus [5, 75, 76]. The association between CHDs and EF is thought to be the result of a complex interplay between various endogenous or exogenous factors, such as low oxygen saturation, abnormal cerebral blood flow, and the use of cardiopulmonary bypass during surgery, which in turn affect early brain development [77–82]. The various factors differ between different types of CHD as their hemodynamic impact varies, and as the type and magnitude of intervention depends on the nature and severity of the CHD. Alternatively—or additionally—, the concurrent presence of a CHD and neurodevelopmental impairments may be explained by pleiotropy; that is, the same pathogenic genetic variant underlying the CHD may also affect brain development [83–86]. Figure 2 shows a simplified illustration of the various potential causal pathways between CHD and EF impairment.

In line with the hypothesis that pleiotropy explains the concurrent presence of a CHD and neurodevelopmental impairments, studies with individuals with 22q11DS have reported that although differences in cortical thickness were related to CHDs [87, 88], no effect of CHDs on the developmental trajectory of EF was observed [87]. Additionally, several studies have reported an absence of evidence for an effect of CHDs on various components of cognitive functioning in 22q11DS (e.g., [34, 89–95]. For example, Zhao et al. [96] found an effect of deletion size, but not of CHD, on IQ in a sample of more than 1000 individuals with 22q11DS. For EF specifically, one study investigated whether the presence of CHDs was associated with EF performance in four groups of 8- to 14-year-old children: children with 22q11DS with and without CHD, children with a CHD without a syndromic origin (CHD-only), and TD children [97]. The 22q11DS groups did not differ from one another and both performed worse than the TD and the CHD-only group on all EF measures. Notably, in contrast to previous findings in non-syndromic CHD samples, the latter two groups did not differ from each other.

Taken together, these findings suggest that the impact of the 22q11.2 deletion exceeds the hypothesized impact of CHD. This is further supported by findings in another pathogenic variant, Down syndrome (trisomy 21), in which CHDs are also common. In this population, CHDs were largely unrelated to EF performance [98], although a small impact of CHDs on neurodevelopmental outcomes may be present during the preschool age [99, 100]. In 22q11DS, it is yet unknown whether CHDs is related to EF skills at such a young age.

### Current study

In the current study, we compared EF performance of 44 preschoolers with 22q11DS (3.0–6.5 years) to 81 TD peers. The first aim of this study was to provide an overview of EF abilities of preschool-aged children with 22q11DS. We administered measures of visual selective attention, visuospatial working memory, and broad EF. Based on the literature discussed above, we hypothesized lower performance of the children with 22q11DS in comparison with TD controls. Given the mixed findings on WM in the literature and the scarcity of studies on selective attention, we had no specific hypotheses, although WM skills may be a relative strength of children with 22q11DS. Additionally, we investigated the relations between the different EF tasks as a first step in exploring the overall EF profile in this young age-group. As selective attention has been proposed to be a prerequisite for further EF development [27], we expected it to be significantly correlated with both the working memory and the broad EF task. We also considered the effect of age, IQ, and socioeconomic status.

The second aim of this study was to explore the effect of a hemodynamically significant CHD (HS-CHD) on EF performance in preschoolers with 22q11DS. Based on studies in older children or adults with 22q11DS (e.g., [87, 97]), we hypothesized that the impact of a CHD on EF as observed in the general population [8, 9], is overshadowed by the impact of the genetic deletion [79, 86]. We also considered the possibility that a CHD would explain some variance in the EF performance of our participants with 22q11DS, as previous work in a different pathogenic variant (trisomy 21) suggests that the impact of CHDs may be particularly meaningful in the preschool age [98].

## Methods

### Participants

A total of 125 children, of which 44 children with 22q11DS and 81 TD controls, participated in this study as part of a larger prospective study (‘3T project’) investigating children’s language, cognitive, and behavioral development. The study was approved by the Medical Research Ethics Committee of the University Medical Centre Utrecht, the Netherlands (CCMO registry nr. NL63223.041.17). All parents of the participating children provided written informed consent. Children were recruited between November 2018 and November 2019. Inclusion criteria were (1) monolingual Dutch, (2) aged between 3.0 and 6.5 years, and (3) no documented hearing loss (> 35 dB).

For children with 22q11DS, an additional inclusion criterium was (4) a 22q11DS deletion confirmed by genetic testing (see Additional file 1: Appendix A). Children with 22q11DS were recruited through the national multidisciplinary outpatient clinic for children with 22q11DS (University Medical Centre Utrecht) and the Dutch 22q11DS patient support group (Stichting Steun 22Q11). One participant was recruited via a different medical center in the Netherlands (see [101] for a flow-chart of participant in- and exclusion). For TD children, an additional inclusion criterium was (4) no history of developmental concerns and no family history of language impairment.^1^ TD children were recruited through day-care centers and elementary schools throughout the Netherlands. In some cases, they were recruited from the same schools that were attended by children with 22q11DS who participated in this study. Other schools were approached separately by the research team. Sample characteristics are presented in Table 1.

**Table 1:** Sample characteristics of the children with 22q11DS (*n* = 44) and the TD children (*n* = 81)

	22q11DS	TD	
*N* female (%)	19 (43%)	45 (56%)	*χ*^2^(1) = 1.29, *p* = 0.26, *V* = 0.12
Mean age (SD)	4.9 (1.0)	4.7 (0.9)	*t*(79.229) = 1.63, *p* = .21, *g* = 0.21
Range (year;month)	3;1–6;5	3;0–6;6	
Mean IQ^a^ (SD)	80.2 (11.7)	105.6 (13.4)	*t*(93.989) = 117.07, *p* < 0.001, *g* = 1.98
Range	50–103	78–139	
Mean SES^b^ (SD)	6.4 (1.8)	7.8 (1.3)	*t*(69.007) = 20.96, *p* < 0.001, *g* = 0.94
Range	2–9	3.5–9	
Mean PPVT^c^ (SD)	83.7 (14.0)	108.5 (11.9)	*t*(72.374) = 24.74, *p* < 0.001, *g* = 1.96
Range	55–114	79–145	
Mean CLI^c^ (SD)	70.8 (12.2)	105.7 (13.3)	*t*(73.420) = 34.89, *p* < 0.001, *g* = 2.69
Range	55–102	79–133	

*Abbreviations*: *22q11DS* 22q11.2 deletion syndrome, *CLI* Core Language Index, *IQ* Intelligence Quotient, *PPVT* Peabody Picture Vocabulary Test, *SD* standard deviation, *SES* socio-economic status, *TD* typically developing

^a^For children with 22q11DS, intelligence quotient (IQ) scores were obtained from medical records or school. These IQ tests were administered by a licensed psychologist in the context of formal cognitive assessments. Two children with 22q11DS had no recent IQ scores. For one of these children a trained researcher from the current study administered the shortened version of the Wechsler Non-Verbal (WNV) [102]. For TD children, the shortened version of the WNV was administered by one of the trained researchers from the current study. A valid IQ score could not be obtained for one TD child after repeated non-compliance to the task instructions

^b^Socioeconomic status was indexed by the average education level of both parents, ranked on a 9-point scale reflecting the Dutch educational system, ranging from 1 ‘not completed primary education’ to 9 ‘university degree’. The average both parents was taken unless the child came from a single parent household (22q11DS *n* = 5; TD *n* = 0). SES is missing for one TD child, as parents declined to answer

^c^The PPVT-III-NL is a measure of receptive vocabulary and the CLI from the CELF Preschool-2-NL is an index score that reflects overall language ability. Both are normed with a mean of 100 and an SD of 15. For a detailed description of the language profile of the 22q11DS sample see [101] and [103]. For the 22q11DS group: PPVT *n* = 42 and CLI *n* = 36; for the TD group CLI *n* = 80

### Cardiac phenotype

For the children with 22q11DS, the presence of any type of CHD, hemodynamic significance of the CHD (HS-CHD), and surgical intervention were assessed by a pediatric cardiologist based on review of medical records (*n* = 42) and parental report (*n* = 2^2^). Twenty-five children with 22q11DS had some type of CHD. There were 13 children with only a single CHD diagnosis, while 12 children had multiple cardiac diagnoses. The most common CHD was ventricular septal defect (*n* = 16). Children with HS-CHD (*n* = 16) were compared with all other children (*n* = 28) for the purpose of our analysis, as these types of CHD likely have the largest impact on early brain development (see Fig. 2). All children in the HS-CHD group had undergone surgery, all but one with cardiac pulmonary bypass. See Additional file 1: Appendix B for a more detailed description of the cardiac phenotypes of the sample.

Parents of TD children were asked if their child had CHD, but none of the parents reported that this was the case.

### Procedure

Behavioral assessment of the EF tasks took place at the child’s school or day-care center and consisted of two sessions of 45 min each, which were on average 5 (SD = 3, range 0–14) days apart. Both sessions were always conducted by the same trained researcher. EF tasks were mixed with other cognitive and language tasks and administered in a fixed order. Parents filled in online questionnaires regarding demographic information and their child’s development.

### Outcome measures

#### Selective attention

**Fig. 3:**
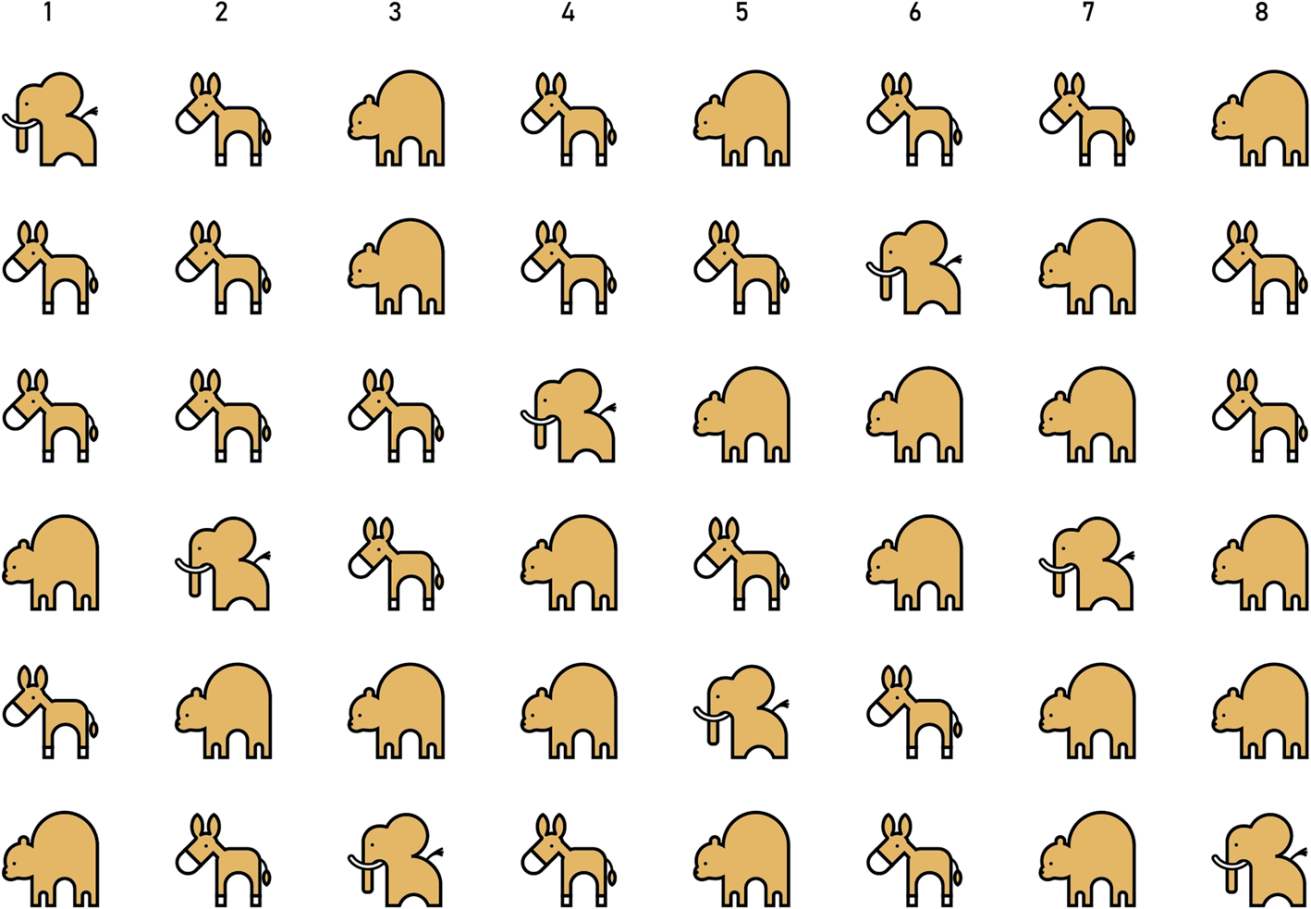
Search display 1 of the SA task [106]

We used a task developed by Mulder et al. [104] to measure selective attention (SA). Children were instructed to search elephants among distractors (donkeys and bears) in four displays, which differed in the number and/or size of the animals. The search displays were presented on a 15.6-inch screen on a HP ProBook 450 G5 Notebook laptop using E-Prime 2.0 [105]. Children were instructed to point to the elephants they had found. To minimize working memory load, targets detected by the child were crossed with a blue line. Each display was presented for 40 s. The first two displays contained 40 distractors and 8 targets (6 rows, 8 columns; see Fig. 3). The third display contained 64 distractors and 8 targets (9 rows, 8 columns), and the fourth display contained 195 distractors and 9 targets (12 rows, 17 columns). Children were not informed of the number of targets present in any display. SA outcome measures were (1) the number of targets found (Hits), (2) the number of incorrect responses (i.e., pointing to distractors; Errors), and (3) the number of repeated responses (i.e., targets already marked as found; Repetitions). These were computed per display, as well as in total for all displays together.

#### Working memory

**Fig. 4:**
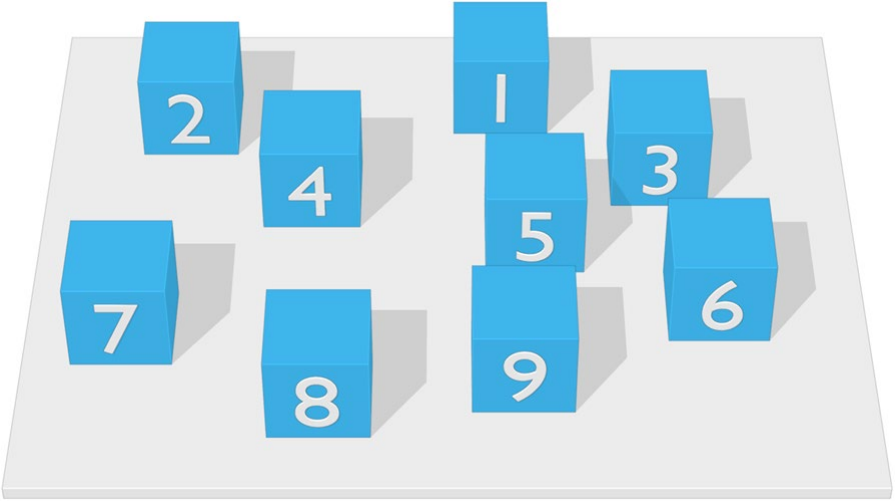
Corsi block task as seen from the perspective of the experimenter. Numbers on the blocks were not visible to the participant. Figure adapted from [114]

The Corsi Block tapping task was administered to gauge visuo-spatial WM skills [107–109]. Children were presented with a white board with nine blue blocks, following the set-up of Kessels et al. [110] (see Fig. 4). We followed the procedure of the Mind Prekindergarten Curriculum [111, 112], as translated into Dutch by Wijnroks et al. [113]. This task has two conditions with two tests each.

In the forward (FW) condition, the child was instructed to tap the blocks in the same order as the experimenter. The experimenter accompanied these instructions with the corresponding actions. After four practice trials, the first test started with a sequence of two blocks. If the child copied the sequence correctly, the experimenter moved on to the next sequence length. If the response was incorrect, the experimenter showed a second trial with a different sequence of the same length. If the child failed to copy this sequence, the test was terminated. The Backward (BW) condition was administered in the same way, except that the child had to tap the sequences in reverse order. The BW condition requires the information stored to be manipulated (i.e., the sequence of the items must be reversed by the participant) and is therefore considered a more valid measure of working memory than the FW condition, for which information merely has to be reproduced [115]. The sequences increased in length with one block each time with a maximum of nine blocks in the FW condition and six blocks in the BW condition. All sequences were predetermined and the same for all children. Of the two tests, the longest successfully copied sequence length was taken as the outcome measure. In the FW condition, children who successfully completed the practice items but did not repeat any of the test items correctly were awarded a score of 1. Children who did not understand the BW condition, but who successfully completed at least one trial of the FW condition, thereby demonstrating comprehension of the task instructions, were awarded a score of 1 for the BW condition.

#### Broad EF

The Head-Toes-Knees-Shoulders (HTKS) [116, 117] is a task gauging a broad scope of EF abilities. The HTKS was developed as an ecologically valid measure of multiple aspects of EF. The HTKS is considered a broad EF measure, as it requires the child to keep the rules of the game active in working memory during the task, to use these rules to select correct responses, and to inhibit a natural, but incorrect response, while directing their attention to the experimenter. We followed the procedure of the Mind Prekindergarten Curriculum [111, 116, 117] as translated into Dutch by Wijnroks et al. [113]. The task consists of two parts.

In the first part, children were asked to point to their head and to their toes (HT condition). Children were told that they were going to play a ‘weird’ game and were instructed to do the opposite of what the experimenter told them to do. So, if the experimenter told them to point to their toes, they had to point to their head and vice versa. All instructions were accompanied by the corresponding movements by the experimenter. The child was encouraged to copy these movements during the instructions. After 4 practice trials, 10 test trials were administered. *Head* and *toe* trials were administered in a fixed non-alternating order. For a correct response, children were awarded 2 points. If a child made a clear self-correction and thus eventually responded correctly, they were awarded 1 point. For incorrect responses, they were awarded 0 points. Thus, for the first part, a total of 20 points could be obtained. Scores were only considered valid if children responded correctly to at least two practice trials. Otherwise, their score was marked as missing as it could not be reliably established whether children either did not understand the task instructions or could not perform the task.

If a child obtained more than 10 points in the first part of the task, the second part of the task was administered. Children were asked to point to their knees and to their shoulders (KS condition). Children were again instructed to do the opposite of what the experimenter told them to do. After four KS practice trials, HT trials were added. Following the same procedure as for the HT condition, 10 test trials were administered and scored.

The task was filmed and also scored by a second researcher. In case of discrepancies between the scores by the experimenter and the second researcher, final scores were determined through a consensus procedure (22q11DS:* n* = 1; TD: *n* = 4). In addition to the accuracy score, the number of self-corrections was also registered.

#### Data analyses

Data was prepared and analyzed using R version 4.0.2 [118] and IBM SPSS 27.0 (2020). As not all participants were able to complete all tasks, analyses always included the maximum number of available participant scores. Parametric results are reported unless non-parametric tests were required and showed different outcomes than parametric tests. Comparison of demographic variables between the groups and between children with and without complete task data was done using Welch’s *t* test [119]. All significance tests were two-tailed with an *α* of 0.05. No formal statistical analysis was performed when the majority of children had incomplete task data, as the outcomes would likely be biased and not give an accurate reflection of the capabilities of the respective populations.

The first aim of the current study was to provide an EF profile of young children with 22q11DS as compared to a TD control group. Incomplete task data was considered informative, as it is indicative of a child’s level of functioning. *χ2* tests were used to compare the distributions of children with and without complete task data between the groups. Prior to the primary analysis, correlations were used to determine the relationship of different outcomes of the same task. As each task has multiple outcome measures, we report Pillai’s trace values from multivariate analysis of variance (MANOVA) which corrects for multiple testing. Greenhouse–Geisser corrections were used when Sphericity could not be assumed. For the MANOVAs, *group* was taken as the independent variable. For the SA task, the dependent variables were *hits*, *errors*, and *repetition*s; for the WM task the dependent variables were longest *span* in the *forward* (*FW span*) and in the *backward* condition (*BW span*); and for the broad EF task, it was the accuracy *score* and *self-corrections (SC)* for both part I (HT) and part II (KS). Additionally, for the SA task, a repeated measures MANOVA was used to investigate whether the groups differed on performance (*Hits*, *Errors*, *Repetitions*) with increasing complexity (*display*). Finally, to gain more insight into the overall EF profile of both groups of children, Pearson bivariate correlations were used to investigate the relations between the various EF outcomes.

The second aim of the current study was to explore the effect of CHD on EF performance in children with 22q11DS. Using the same analyses for the comparison with TD children, children with 22q11DS with HS-CHD were compared to children with 22q11DS without HS-CHD. As many factors related to CHD may impact early cognitive development (see “Congenital heart defects” section), we ran sensitivity analyses [120]. In these sensitivity analyses, we used different CHD grouping criteria: (1) the presence of any type of cardiac anomaly (*n* = 25), and (2) having undergone cardiac surgery^3^ (*n* = 18). Sensitivity analyses were the same as the main analyses with regard to models and tests used.

In all analyses, *age* was used as a covariate, as age is correlated with the outcome measures but unrelated to the independent variable *group* (see Table 1 and Additional file 1: Appendix C). *Socioeconomic status (SES)* was also considered as a covariate, as there was a significant difference in *SES* between the groups (see Table 1) and because previous research has suggested that SES might affect EF outcomes in TD children [121] (but see [122, 123] for 22q11DS). As differences in IQ are inherent to the groups, IQ was not considered as a covariate in the group comparisons with the TD controls [124, 125]. It was, however, used as a covariate in the CHD analyses and considered in relation to the EF measures in the exploratory correlation analyses. These correlations between the EF tasks and age, SES, and IQ can be found in Additional file 1: Appendix C. Only covariates that had a significant effect on the outcome are reported.

## Results

### Selective attention

#### Descriptives and task completion data

Selective attention outcomes are reported in Table 2. Two children with 22q11DS of 4.6 and 3.3 years old could not complete the SA task due to low mental age and high levels of inattention, respectively. All TD children completed the SA task.

**Table 2:** Results of the SA task for the children with 22q11DS (*n* = 42) and the TD children (*n* = 81)

		Hits			Errors			Repetitions		
		*M*	SD	Range	*M*	SD	Range	*M*	SD	Range
Total	22q11DS	19.95	4.44	10–28	1.88	2.09	0–9	0.48	1.04	0–5
	TD	22.73	3.96	13–31	0.57	1.14	0–6	0.31	0.58	0–2

The maximum number of Hits is 33. There was no maximum number of Errors and Repetitions. For outcomes per display, see Additional file 1: Appendix D

*Abbreviations: 22q11DS* 22q11.2 deletion syndrome, *SD* standard deviation, *TD* Typically Developing

#### Within task correlations SA outcome measures

*Hits* and *errors* were negatively correlated in both the 22q11DS group (*r*(42) =  − 0.36,* p* = 0.018, 95% CI [− 0.60 to − 0.07]) and the TD group (*r*(81) =  − 0.24,* p* = 0.029, 95% CI [− 0.44 to − 0.03]), indicating that children who found more targets made fewer errors. In the 22q11DS group, *repetitions* were not correlated with *hits* (*r*(42) = 0.06,* p* = 0.69, 95% CI [− 0.25–0.36]) or *errors* (*r*(42) = 0.18,* p* = 0.24, 95% CI [− 0.13–0.46]). *Repetitions* were also not correlated with *hits* (*r*(81) =  − 0.06,* p* = 0.59, 95% CI [− 0.28–0.16]) or *errors* (*r*(81) = 0.05,* p* = 0.64, 95% CI [− 0.17–0.27]) in the TD group.

### Group comparisons between the children with 22q11DS and the TD children

**Fig. 5:**
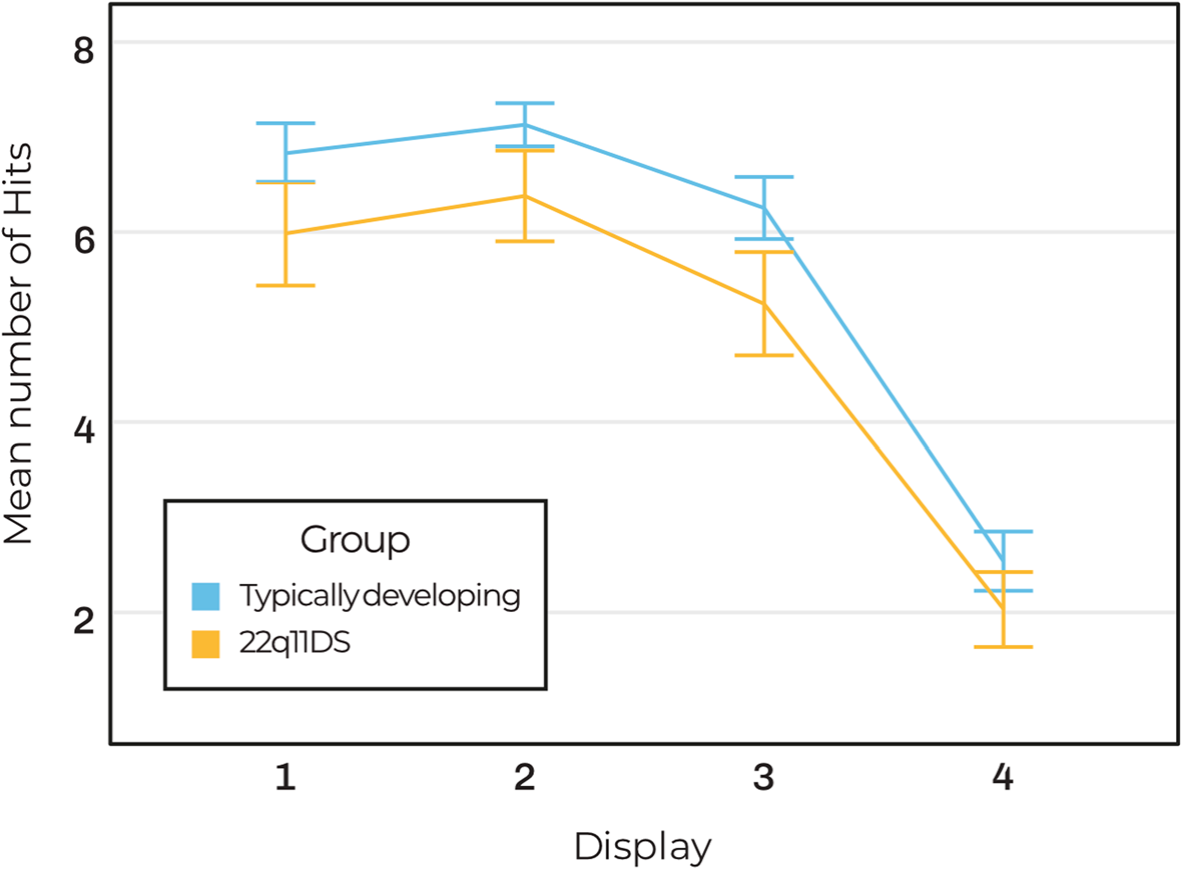
SA task for the children with 22q11DS (*n* = 42) and the TD children (*n* = 81); line chart of the mean number of *hits* per display for each group. Errors bars indicate 95% CI

A repeated measures MANOVA showed that there was an effect of *group* on the SA task (*V* = 0.18, *F*(3, 119) = 8.57, *p* < 0.001, *η*_*p*_^*2*^ = 0.18). Children with 22q11DS had a lower total number of *Hits* (*F*(1, 121) = 12.51, *p* < 0.001, *η*_*p*_^*2*^ = 0.09) and made more *errors* (*F*(1, 121) = 20.44, *p* < 0.001, η_p_^2^ = 0.15) than TD children. There was no difference in the total number of *repetitions* between the groups (*F*(1, 121) = 1.31, *p* = 0.26, *η*_*p*_^*2*^ = 0.011). There was also a main effect of *Display* (*V* = 0.90, *F*(9, 113) = 111.22, *p* < 0.001, *η*_*p*_^*2*^ = 0.90). This effect of *display* was only significant on *hits* (after Greenhouse–Geisser correction) (*F*(2.753, 333.143) = 424.09, *p* < 0.001, *η*_*p*_^*2*^ = 0.78), but not on *errors* (*F*(2.908, 351.863) = 1.52, *p* = 0.21, *η*_*p*_^*2*^ = 0.01) or *repetitions* (*F*(2.549, 308.382) = 1.44, *p* = 0.24, *η*_*p*_^*2*^ = 0.01). This shows that the number of *hits* decreased with increasing *display* complexity. There was no interaction between *group* and *display* (*V* = 0.06, *F*(9, 113) = 0.85, *p* = 0.57, *η*_*p*_^*2*^ = 0.06), indicating that this effect of *display* was similar across both groups (see Fig. 5). These findings did not change when *age* and *SES* were entered as covariates. Only *age* was a significant covariate (*V* = 0.32, *F*(3, 118) = 18.52, *p* < 0.001, *η*_*p*_^*2*^ = 0.32), resulting in a larger effect size for *group* (*η*_*p*_^*2*^ = 0.27). These results should be interpreted with caution as the assumption of homogeneity of covariance matrices was violated.

### Working memory

#### Descriptives and task completion data

Working memory outcomes per group are reported in Table 3. In the 22q11DS group, eight children were unable to complete the FW and BW condition. In the TD group, three children were unable to complete the FW and BW condition, and one additional child was unable to complete the BW condition. Given the small samples and unequal sample sizes, we only describe the differences on demographic variables between children with complete and incomplete task data per group, but we did not carry out statistical analyses for these comparisons.

**Table 3:** Results of the WM task of the children with 22q11DS and the TD children

		*N*	*M*	Median	SD	Range
FW span	22q11DS	36	2.86	3.0	0.83	1–5
	TD	78	3.51	4.0	0.94	1–6
BW span	22q11DS	36	1.81	2.0	0.82	1–3
	TD	77	2.43	2.0	1.14	1–6

The maximum span for the FW condition is 9 and 6 for the BW condition

*Abbreviations*: *22q11DS* 22q11.2 deletion syndrome, *BW* backward, *FW* forward, *SD* standard deviation, *TD* typically developing

The children with 22q11DS who did not complete one or both conditions from the WM task included five boys and three girls. They were younger (*n* = 8; *M*_age_ = 3.6, SD = 0.5) than children with 22q11DS with complete task data (*n* = 36; *M*_age_ = 5.2, SD = 0.9). Their IQ score (*M* = 71.7, SD = 11.4, range 50 to 81) appeared lower than that of children with complete task data (*M* = 81.9, SD = 11.1, range 55 to 103), while their SES (range 2–8.5) appeared similar. The TD children who did not complete one or both conditions from the WM task included two boys and two girls. They were younger (*n* = 4; *M*_age_ = 3.5, SD = 0.2) than TD children with complete task data (*n* = 77; *M*_age_ = 4.7, SD = 0.9). They had average IQ scores (range 96–109) and did not appear to differ in SES (range 8–9) from the rest of the group.

#### Within task correlations WM outcome measures

The *FW span* and *BW span* were strongly correlated in the TD children (*r*(77) = 0.58, *p* < 0.001, 95% CI [0.41–0.71]). In children with 22q11DS, *FW span* and *BW span* showed a trend towards a moderate correlation, but this did not reach statistical significance (*r*(36) = 0.29, *p* = 0.083, 95% CI [− 0.04–0.57]).

#### Group comparisons between the children with 22q11DS and the TD children

There was a significant effect of *group* on the WM task (*V* = 0.13, *F*(2, 110) = 6.77, *p* < 0.001, *η*_*p*_^*2*^ = 0.13). Children with 22q11DS had a shorter *FW span* (*F*(1, 111) = 14.93, *p* < 0.001, *η*_*p*_^*2*^ = 0.12) and shorter *BW span* (*F*(1, 111) = 8.63, *p* = 0.004, *η*_*p*_^*2*^ = 0.07) than TD children. These findings did not change when *Age* and *SES* were entered as covariates. Only *age* was a significant covariate (*V* = 0.41, *F*(2, 109) = 37.61, *p* < 0.001, *η*_*p*_^*2*^ = 0.41), resulting a larger effect size for the effect of *group* (*η*_*p*_^*2*^ = 0.31).

### Broad EF

#### Descriptives and task completion data

Broad EF outcomes per group are reported in Table 4. However, data of the broad EF task was incomplete for a substantial number of participants. There were relatively more children with incomplete task data in the 22q11DS group (*n* = 35/44, 80%) than in the TD group (*n* = 23/81, 28%; *χ*^*2*^(1) = 30.0, *p* < 0.001, *V* = 0.49).

**Table 4:** Results of the broad EF task of the children with 22q11DS and the TD children

			*N*	*M*	Median	SD	Range
Part 1–HT	*Score*	22q11DS	12	11.8	16	7.7	0–20
		TD	65	16.6	18	4.5	0–20
	*SC*	22q11DS	12	2.0	2,5	1.6	0–4
		TD	65	1.2	1	1.1	0–4
Part 2–KS	*Score*	22q11DS	10	10.0	11	6.0	0–18
		TD	58	11.7	13,5	6.0	0–19
	*SC*	22q11DS	10	1.8	1	1.7	0–5
		TD	58	1.8	2	1.7	0–4

The maximum for Score is 20 and for SC is 10

*Abbreviations*: *22q11DS* 22q11.2 deletion syndrome, *HT* Head-Toes, *KS* Knees-Shoulders, *SC* self-correction, *SD* standard deviation, *TD* typically developing

In the 22q11DS group, 31 children were unable to complete the HT part and one child had missing data due to a task administration error. The latter child did have data for the KS part. Three additional children were unable to complete the KS part of the task. Children with 22q11DS missing one or both conditions from the HTKS task were younger (*M* = 4.7, SD = 1.0) than children with 22q11DS who completed the task (*M* = 5.8, SD = 0.3; *p* < 0.001). There was no difference between these groups in sex distribution (*p* = 0.40), SES (*p* = 1.0), or IQ scores (*p* = 0.55). In the TD group, 16 children were unable to complete the HT condition of the task, and 7 additional children were unable to complete the KS condition. TD children missing one or both conditions from the HTKS task were younger (*M* = 3.7, SD = 0.6) than TD children who completed the task (*M* = 5.0, SD = 0.7; *p* < 0.001). There was no difference between these groups in sex distribution (*p* = 0.91), SES (*p* = 0.19), or IQ scores (*p* = 0.08). See Additional file 1: Appendix E for a detailed description and the complete statistics.

Since a substantial number of participants had incomplete task data for the HTKS, no formal statistical analyses were performed.

### Exploratory correlations–EF profile

To explore the EF profile of the childen with 22q11DS as compared to that of TD children, we examined the correlations between the SA and WM outcomes per group. The HTKS was excluded from these analyses due to the large amount of missing data.

There were several significant correlations between the SA task and the WM task (see Table 5). In the TD group, SA hits was positively correlated with both the Corsi FW and BW scores, indicating that TD children who found more targets in the SA task also had longer WM span scores. These correlations were not significant in the 22q11DS group. SA errors was negatively correlated with the Corsi FW in the children with 22q11DS and with the Corsi BW in the TD children.

**Table 5:** Correlations between the SA task and WM task for the children with 22q11DS and the TD children

			WM forward				WM backward		
		*n*	*r*	*p*	95% CI	*n*	*r*	*p*	95% CI
SA hits	*22q11DS*	36	0.29	0.082	− 0.04–0.57	36	0.17	0.32	− 0.17–0.47
	*TD*	78	**0.59**	** < 0.001**	0.36–0.68	77	**0.47**	** < 0.001**	0.27–0.63
SA errors	*22q11DS*	36	− **0.50**	**0.002**	− 0.71 to − 0.21	36	0.11	0.53	− 0.23–0.42
	*TD*	78	− 0.04	0.71	− 0.26–0.18	77	− **0.23**	0.042*	− 0.44 to − 0.00
SA repetitions	*22q11DS*	36	− 0.08	0.63	− 0.40–0.25	36	0.18	0.29	− 0.48–0.16
	*TD*	78	0.07	0.51	− 0.15–0.29	77	0.05	0.68	− 0.27–0.18

Significant correlations are in bold

*Abbreviations*: *22q11DS* 22q11.2 deletion syndrome, *CI* confidence interval, *SA* selective attention, *SD* standard deviation, *TD* typically developing, *WM* working memory

^*^Spearman’s rho, as these non-parametric outcomes differed from the Pearson correlation (*r*(77) =  − 0.22, *p* = 0.056)

### The impact of hemodynamically significant CHD on EF in 22q11DS

#### Descriptives and task completion data

Task completion, age, SES, and sex distribution were not significantly different between the children with and without hemodynamically significant CHD (HS-CHD) (*p* = 0.94, *p* = 0.76,* p* = 0.39, and* p* = 0.57, respectively). However, there was a trend towards a lower IQ for the children with HS-CHD (*M* = 75.4, SD = 12.2) as compared to those without HS-CHD (*M* = 82.9, SD = 10.7; *p* = 0.056). See Additional file 1: Appendix F for a detailed description and the complete statistics. Outcomes per EF task of both groups are displayed in Table 6.

**Table 6:** EF results of the children with 22q11DS with and without HS-CHD

	HS-CHD	*n*	*M*	Median	SD	Range
SA hits	*Yes*	15	19.5	21	4.3	13–25
	*No*	27	20.2	21	4.6	10–28
SA errors	*Yes*	15	2.8	3	2.4	0–9
	*No*	27	1.4	1	1.7	0–5
SA repetitions	*Yes*	15	0.9	0	1.6	0–5
	*No*	27	0.3	0	0.5	0–2
WM forward	*Yes*	13	2.8	3	0.9	1–4
	*No*	23	2.9	3	0.8	1–5
WM backward	*Yes*	13	1.6	2	0.7	1–3
	*No*	23	1.9	2	0.9	1–3

The maximum of SA Hits is 33, that of WM forward is 9, and that of WM backward is 6. SA errors and SA repetitions have no maximum

*Abbreviations*: *HS-CHD* hemodynamically significant congenital heart defects, *M* mean, *SA* selective attention, *SD* standard deviation, *WM* working memory

### Group comparisons between the children with 22q11DS with and without HS-CHD

There was no effect of HS-CHD on the SA task (*V* = 0.16, *F*(3, 38) = 2.45, *p* = 0.079, *η*_*p*_^*2*^ = 0.16). Covariates *age*, *SES*, and *IQ* were not significant and did not change these findings. Results should be interpreted with caution as the assumption of homogeneity of covariance matrices was violated.

There was no effect of HS-CHD on the WM task (*V* = 0.03, *F*(2, 33) = 0.55, *p* = 0.58, *η*_*p*_^*2*^ = 0.03). These findings did not change when *age*, *SES*, and *IQ* were entered as covariates. *Age* was a significant covariate (*V* = 0.26, *F*(2, 29) = 6.36, *p* = 0.005, *η*_*p*_^*2*^ = 0.31), but did not change the effect of HS-CHD.

 All sensitivity analyses showed similar results (see Additional file 1: Appendix G). The only effect was observed in the comparison between children with any type of cardiac anomaly (CA) and those without. Children with CA made more SA errors, but the distribution of errors was skewed and should be interpreted with caution.

## Discussion

The aim of the current study was twofold. The first aim was to describe the executive functioning (EF) profile of 3.0- to 6.5-year-old children with the 22q11.2 Deletion Syndrome (22q11DS) and to compare this to that of typically developing (TD) peers. The second aim was to examine the relation between EF abilities and the presence of a hemodynamically significant congenital heart defect (HS-CHD) in children with 22q11DS. EF was assessed with behavioral tasks measuring visual selective attention (SA), working memory (WM), and a task gauging broad EF abilities.

### Selective attention

To our knowledge, this is the first study to investigate SA in young children with 22q11DS. Our results show that visual SA is impaired in children with 22q11DS, as indicated by the fact they found 14% fewer targets and made more than three times as many errors as their TD peers. The finding of impaired SA is in line with outcomes in older children with 22q11DS [48], and with more general findings of impaired attentional functioning in these children (e.g., [42, 44, 46, 47, 126]). A previous study looking at visual attention showed that children with 22q11DS were more sensitive to task load than TD peers as shown by an increase in errors with increasing task load [44]. However, in our study, there was no evidence for a difference in response to increased task complexity between the children with 22q11DS and the TD children. That is, when the number of distractors in the display increased, the number of targets found decreased and the number of mistakes made increased roughly equally for both groups. It should be noted that the number of errors as well as repetitions were skewed due to their low occurrence and limited variance, so the results of the analyses with these outcomes should be interpreted with caution.

As SA is considered an important precursor of later EF abilities [27, 32], this apparent impairment in SA suggests that EF impairment likely emerges already very early on in children with 22q11DS. Pending replication in other studies, this finding provides a rationale for early intervention aimed at strengthening SA in young children with 22q11DS as a possible means to support further EF development [127–129].

### Working memory

Based on a recent review of previous studies that showed mixed outcomes regarding working memory abilities in school-aged children and adolescents with 22q11DS [7], we considered it likely that WM could be relatively spared. Our results, however, show that visual WM abilities of preschoolers with 22q11DS are weaker than those of TD peers. Children with 22q11DS had a forward span that was 23% and a backward span that was 34% shorter than TD children on the Corsi block tapping task. Another group conducted two studies with the same sample of children with 22q11DS in which they administered the forward condition of the Corsi task. The backward condition of the Corsi was not administered. These studies, however, showed diverging outcomes. One study reported that the sample of 6- to 12-year-old children with 22q11DS (*n* = 25) performed worse than TD controls [34], while the other study reported that there was no difference on the Corsi forward span between the groups [33]. This difference is likely due to the inclusion of additional groups in the statistical analyses performed in the latter study. A study using a task similar to the Corsi forward condition showed that children with 22q11DS (6–15 years old) made more mistakes than the TD controls [130]. Our results support the outcomes of Wong et al. [130] and De Smedt et al. [34], and are in line with studies using different tasks to gauge WM skills [35, 37–40] and imaging studies that showed aberrant functional activity in brain areas associated with WM [131–133]. This strengthens the hypothesis that visuospatial WM is impaired in children with 22q11DS. The current study is the first to provide evidence that these impairments are probably already present at a young age. More research with young children with 22q11DS is necessary to corroborate our results.

Additionally, the Corsi forward span and Backward span were significantly correlated in the TD children, in line with previous research [107, 134], but notably this was not the case in the children with 22q11DS. This may be partly due a lack of power, or, alternatively represents an aberrant developmental trajectory of WM in 22q11DS.

The outcomes of the current study regarding WM are limited to the visual domain. Future research should also investigate whether verbal WM is impaired at this young age, as research in primary school-aged children with 22q11DS found that verbal WM may be a relative strength [33, 34]. This may, however, be challenging as many of verbal WM tasks, such as the Digit Span, are not well suited for young children.

### Broad EF

Results from the broad EF task were limited by the fact that a substantial number of children was not able to complete this task. This task might have been too difficult as it requires children to understand complex instructions, retain these instructions in their working memory, inhibit automatic responses and maintain attention to listen to the experimenter [117, 135–137]. Visual inspection of the data from children who could complete the task suggests that the children with 22q11DS did not perform as well as the TD children.

A majority of TD children, but only a small group of children with 22q11DS were able to complete the task. There was no difference in chronological age between the two groups and in both groups, children missing one or both conditions from the HTKS task were significantly younger than children who completed the task. The fact that children who could not complete the task are significantly younger, could hint at either a ‘developmental deficit’ or a ‘developmental lag’ [138], but longitudinal data is needed to investigate this. The fact that there was no difference in intelligence quotient (IQ) scores between children with 22q11DS with and without complete task data suggests that chronological age and other factors play an equally significant or more important role in performing this task than intellectual level. This could be verified by research administering this task to older children with 22q11DS in comparison to both younger mental-age matched TD children and chronologically age-matched TD peers.

### EF profile

Our results suggest that the different components of EF may be less strongly interrelated in 22q11DS compared to TD peers. Our findings in TD children support the model of Garon et al. [27] and are in line with previous research showing that selective attention is related to WM skills [32]. In contrast to the TD group, selective attention in children with 22q11DS was not related to either WM outcome. A moderate correlation between the SA task and the Corsi forward emerged in the 22q11DS group, but this did not reach statistical significance. This may be explained by the small 22q11DS sample and therefore insufficient power to identify these correlations. Additionally, the number of errors in the selective attention task was negatively correlated with only the forward condition of the Corsi task in children with 22q11DS, but negatively correlated with the Corsi Backward in TD children. This indicates that children with 22q11DS who made more errors in the selective attention task had lower Corsi forward scores, while TD children who made fewer errors had lower Corsi Backward scores. A possible explanation for this difference is that the ability to perform well on the backward condition builds upon the ability to perform well on the forward condition, creating a developmental shift in the relation between these abilities. Hypothetically, it could be that children with 22q11DS are lagging behind in their development, resulting in an association between selective attention and the less complex WM task condition but, in contrast to TD children, not on the more advanced condition.

Our results are in line with findings in older children and adults with 22q11DS. A recent longitudinal study with older children and adults with 22q11DS (8–35 years) found that all measures of attention and WM were correlated, but that, compared to the TD group, there were fewer correlations between various EF components in the 22q11DS group [46]. Additionally, studies with older children and adults have suggested atypical development of various, but not all EF components [53, 139]. We had planned to collect longitudinal data but were unable to do so due to the COVID-19 pandemic. Future longitudinal research including preschoolers are needed to provide insight in the development and interrelatedness of the early EF profile of children with 22q11DS.

### Congenital heart defects

Previous research has related the presence of congenital heart defect (CHD) to impaired EF in children with non-syndromic CHD [8, 9]. However, this negative impact of CHD on EF abilities may be less clear or even absent in children with 22q11DS [87, 97]. Our results are in agreement with the latter, as we observed no differences in EF abilities between children with 22q11DS and hemodynamically significant CHD (HS-CHD) and children with 22q11DS without HS-CHD in this study. This supports the hypothesis that EF impairments are not (solely) the result of CHD-related procedures. The absence of an effect of HS-CHD on EF in our sample could be explained by the hypothesis that the observed concurrence of CHD and impaired EF is caused by the underlying genetic defect, which leads to CHD but also directly impacts neurodevelopment [83, 84, 86]. It is also possible that there is in fact an effect of surgery and anesthesia or altered oxygenation, but that the direct impact of the 22q11.2 deletion on the brain and cognitive functioning exceeds the hypothesized impact of CHD-related factors.

Sensitivity analyses using different grouping criteria for CHD showed similar results. Overall, sensitivity analyses confirm the lack of evidence for a difference in EF abilities between children with 22q11DS with and without CHD.

### Strengths and limitations

This study is the first to focus on EF abilities in young children with 22q11DS. We used different instruments to assess EF, yielding more robust results and the possibility to study the interrelatedness of different findings [19, 140].

The conclusions of this study and the generalizability of the results are mainly limited by the number of children with 22q11DS who could not complete the WM and broad EF tasks. The variation in developmental level in this group and the rapid development of EF at this age made it difficult to select tasks that were suitable to capture the abilities of all children in this study, including the TD controls. We therefore consider reporting on task incompletion informative and important for transparency. The use of the HTKS had several limitations that could explain the poor task completion of the 22q11DS group. First, although the responses required from children are non-verbal, the instructions are verbal and complex. Children with 22q11DS have impaired language comprehension (e.g., [141–143]), and this also holds for the 22q11DS sample in this study [101, 103]. Children with 22q11DS may not fully understand instructions due to their lower language level and limited working memory abilities [7]. Furthermore, the HTKS was recently revised, as it was suggested that the planning of gross motor movements may be challenging and because disobeying the experimenter goes against the social expectations that children have [144]. As children with 22q11DS also have motor problems (e.g., [145]) and have difficulties with social cognition and pragmatic abilities [146, 147], this may further disadvantage them. For the WM task, verbal instructions were also used but these are less complex than those of the HTKS. Nevertheless, impaired language comprehension may have contributed to some children's inability to complete the task.

The SA task was completed by 95% of children with 22q11DS and all TD children, thereby allowing us to confidently conclude that SA is impaired in young children with 22q11DS. Nevertheless, task performance may have been influenced by visuo-motor impairments, which have been reported in children with 22q11DS ([145, 148–150], but see [45]). Future studies looking at EF should account for impairments in visuo-motor processing and speed.

A strength of this study is our relatively large sample of children with 22q11DS within a narrow age range, allowing us to draw more robust conclusions, given the rapid development at this age. Our 22q11DS sample seems to be representative of the 22q11DS population when looking at phenotypical presentation [5]. Nevertheless, our generalizability may be limited by the fact that children were recruited through medical centers, increasing the chance that our sample consists of children with relatively severe clinical phenotypes.

Although our sample was not large enough to consider the effect of various CHD types and CHD-related factors, we did consider the effect of CHD in various ways, such as grouping based on surgical intervention or hemodynamic significance. This is very important, as CHD is a major somatic symptom associated with the syndrome [5, 71–73] and has also been related to EF abilities in populations with CHD of non-syndromic origin [8, 9]. Large-scale studies, similar to Zhao et al. [96], are needed to further investigate the effect of CHD on EF development in 22q11DS, thereby furthering our understanding of the mechanisms through which CHD affects cognitive functioning. Future studies with both syndromic and non-syndromic populations should look at the additive effects of both genetic variants and CHD-related factors, like surgical intervention, to disentangle their respective impact on early cognitive development.

### Implications

Our results suggest that EF impairments are already present at the preschool age in children with 22q11DS. EF has been shown to be an effective target for intervention [69, 70, 128, 129, 151], but more research is needed to further characterize the early EF profile of young children with 22q11DS and to identify targets for intervention. Early intervention may be crucial, as strengthening EF abilities may be able to mitigate the development of psychopathology or the severity of associated problems [152–155]. This is highly relevant for children with 22q11DS who have a substantially increased risk for psychopathology, including schizophrenia, and developmental disorders such as attenion deficit hyperactivity disorder or autism spectrum disorder [5, 35, 37, 61, 62].

Additionally, our results show that CHD does not appear to increase the risk for EF impairment in early childhood in children with 22q11DS. Although future research is needed to corroborate these findings, this information is useful for parents and clinicians regarding prognosis. More research is needed to determine whether other somatic symptoms experienced by children with 22q11DS, such as hypocalcemia [156, 157] or child-internal or child-external factors [7] pose an additional risk for developing EF problems.

## Conclusion

The present study showed that EF impairments are present at an early age in children with 22q11DS. Both selective attention and working memory abilities are impaired as compared to typically developing peers. Furthermore, different EF components appear to be less interrelated in children with 22q11DS as compared to TD children. Our results do not provide evidence for an effect of congenital heart defects on EF abilities in children with 22q11DS.

## Supplementary Information

**Additional file 1: Appendix A.** Genotype 22q11DS sample. **Appendix B.** Detailed overview CHD characteristics of the 22q11DS sample. **Appendix C.** Correlations demographic variables and EF tasks. **Appendix D.** SA outcomes per display. **Appendix E.** HTKS task completion comparison. **Appendix F.** Task completion CHD group comparison. **Appendix G.** Sensitivity analyses CHD comparison.

## Data Availability

The datasets generated and/or analyzed during the current study are not publicly available due to GDPR compliance and legal and ethical limitations. A limited amount of data can be shared by the corresponding author upon reasonable request.

## Abbreviations

22q11DS: 22q11.2 deletion syndrome
BW: Backward [condition of the Corsi Block Tapping task]
CHD: Congenital heart defect
EF: Executive functioning
FW: Forward [condition of the Corsi Block Tapping task]
HTKS: Head-Toes-Knees-Shoulders task
IQ: Intelligence quotient
MANOVA: Multivariate analysis of variance
SA: Selective attention
SC: Self-correction
SD: Standard deviation
SES: Socioeconomic status
WM: Working memory
WNV: Wechsler non-verbal
TD: Typically developing

## Footnotes

1 Parents were asked in a telephone interview with the researcher if their child or any family members had a language impairment or psychiatric disorders.

2 Medical records could not be obtained due to privacy regulations. For one of these children, parents reported they had regularly visited a multidisciplinary team in an academic hospital and that no CHD was detected. The parents of the other child provided a detailed report of their child’s HS-CHD in a telephone interview with the researcher.

3 There were two cases of children with aberrant subclavian arteries that were surgically corrected (because of esophageal compression), but who did not have HS-CHD.
