# Supplementary material for: Executive functioning in preschoolers with 22q11.2 deletion syndrome and the impact of congenital heart defects
Source: J Neurodev Disord. 2023 May 13;15:15. doi: 10.1186/s11689-023-09484-y (PMC10181926; doi:10.1186/s11689-023-09484-y)
Supplement: Supplementary file 1 — Additional file 1: Appendix A. Genotype 22q11DS sample. Appendix B. Detailed overview CHD characteristics of the 22q11DS sample. Appendix C. Correlations demographic variables and EF tasks. Appendix D. SA outcomes per display. Appendix E. HTKS task completion comparison. Appendix F. Task completion CHD group comparison. Appendix G. Sensitivity analyses CHD comparison. [file 11689_2023_9484_MOESM1_ESM.docx]

**Appendix A – Genotype 22q11DS sample**

All children with 22q11DS were tested with either Copy Number Variation (CNV), Comparative Genomic Hybridization (CGH), or Single Nucleotide Polymorphism (SNP) arrays), Multiple Ligand-dependent Probe Amplification (MLPA), or Whole Exosome Sequencing (WES).

Twenty-eight (64%) children had typical (LCR22A – LCR22D) deletions of ̴ 3 Mb. Eight (18%) children had smaller proximal deletions, five of ̴ 2 Mb (LCR22A – LCR22C), three of ̴ 1,5 Mb (LCR22A – LCR22B), and two children with ̴ 0,5 Mb (LCR22B) deletions. One (2%) child had a 2,7 Mb deletion with a start preceding LCR22A extending to LCR22B. One child (2%) had a ̴ 4 Mb (LCR22B – LCR22F/G) deletion. Three children (7%) had smaller distal deletions: two children with ̴ 0,4 Mb (LCR22C – LCR22D) deletions, and one child had a ̴ 1,1 Mb (LCR22D – LCR22E) deletion. For two children (5%) a 22q11.2 deletion was confirmed by genetic testing, but the exact deletion size could not be obtained from medical records.
 There were three (7%) confirmed familial deletions (1 maternal, 2 paternal) of which one was a typical A-D deletion, and the two others were both distal C-D deletions. For 21 children (48%), the deletion was confirmed de novo. For the other 20 children (45%), deletion origin was unknown.

**Appendix B – Detailed overview CHD characteristics of the 22q11DS sample.**

Table 7. Frequency of CHD, CHD types, and surgical intervention for the children with 22q11DS

|  |  | **N** | **%** | **N  single CHD** | **N multiple CHD** | **N surgical intervention** |
| --- | --- | --- | --- | --- | --- | --- |
| **CHD** | *No* | 19 | 43 | - | - | - |
|  | *Yes* | 25 ^a^ | 56 | 13 | 12 | 18 |
| **CHD diagnosis** | |  |  |  |  |  |
|  | *Ventricular septal defect* | 16 | 64 | 8 | 8 | 10 ^b^ |
|  | *Aberrant subclavian artery* ^c^ | 5 | 20 | 1 | 4 | 5 |
|  | *Right-sided aortic arch* | 4 | 16 | 1 | 3 | 2 |
|  | *Patent ductus arteriosus* | 4 | 16 | 0 | 4 | 3 |
|  | *Stenosis of pulmonary artery* | 3 | 12 | 0 | 3 | 2 |
|  | *Interrupted aortic arch* | 3 | 12 | 3 | 0 | 3 |
|  | *Tetralogy of Fallot* | 3 | 12 | 2 | 1 | 3 |
|  | *Pulmonary valve stenosis* | 2 | 8 | 0 | 2 | 2 |
|  | *Atrial septal defect* | 2 | 8 | 0 | 2 | 2 |
|  | *Truncus arteriosus* | 1 | 4 | 1 | 0 | 1 |
|  | *Double aortic arch* | 1 | 4 | 0 | 1 | 1 |

CHD = Congenital Heart Defect
Note: Multiple diagnoses occurred in 12 children, explaining a sum that is higher than the total. There were no children in the sample who had cardiac defects corrected by means of catheterization only. Four children had catheterization procedures, but these were additional to surgical intervention.
a. Of these, 16 (64%) were hemodynamically significant and 9 (36%) were not.
b. Of these, 4 were isolated cases of VSD.

c. The isolated case concerned the right subclavian artery, while the 4 cases that were accompanied by other cardiac anomalies all concerned the left subclavian artery.

**Appendix C – Correlations demographic variables and EF taks**

There were significant correlations between the EF tasks and demographic variables (see Table 8). *Age* was significantly correlated with all outcomes in the TD group, except for *SA Repetitions*. In the group of children with 22q11DS, *Age* was only significantly correlated with *SA Hits* and *Corsi FW*. Overall, this indicates that older children did better on these EF outcomes as reflected by a positive correlation for all measures except for *SA Errors*, for which a negative correlation was found as lower scores on this outcome indicate better performance. In a non-parametric correlation analysis, *SA Hits* and *Age* were no longer significantly correlated in the 22q11DS group. Assumptions for a parametric analysis are met, but given the small sample size, the results of the Pearson correlation should be interpreted with caution. There were no significant correlations between *SES* and any outcome measure in either group. *IQ* was significantly correlated with SA Hits in TD children but not children with 22q11DS. This positive correlation in TD children indicates that children with higher IQ scores found more targets in the SA task. No other outcome measure was correlated to *IQ* in either group.

Table 8. Correlations between EF outcomes and age, SES and IQ for children with 22q11DS and TD children.

|  |  | **Age** | | | | **SES** | | | | **IQ** | | | |
| --- | --- | --- | --- | --- | --- | --- | --- | --- | --- | --- | --- | --- | --- |
|  |  | *n* | *r* | *p* | *95% CI* | *n* | *r* | *p* | *95% CI* | *n* | *r* | *p* | *95% CI* |
| **SA Hits** | *22q11DS* | 42 | .28 | .077* | -0.04 – 0.54 | 42 | .04 | .80 | -0.27 – 0.34 | 41 | .28 | .072 | -0.03 – 0.54 |
|  | *TD* | 81 | **.70** | **<.001** | 0.57 – 0.80 | 80 | .02 | .87 | -0.20 – 0.24 | 80 | **.31** | **.005** | 0.10 – 0.50 |
| **SA Errors** | *22q11DS* | 42 | -.17 | .28 | -0.45 – 0.14 | 42 | -.16 | .31 | -0.44 – 0.15 | 41 | -.19 | .23 | -0.47 – 0.12 |
|  | *TD* | 81 | -**.32** | **.003** | -0.51 – -0.11 | 80 | .01 | .91 | -0.21 – 0.23 | 80 | -.003 | .98 | -0.22 – 0.22 |
| **SA Repetitions** | *22q11DS* | 42 | .14 | .37 | -0.17 – 0.42 | 42 | .04 | .82 | -0.27 – 0.34 | 41 | -.11 | .49 | -0.40 – 0.21 |
|  | *TD* | 81 | -.17 | .12 | -0.38 – 0.05 | 80 | -.08 | .48 | -0.29 – 0.14 | 80 | .08 | .50 | -0.15 – 0.29 |
| **WM FW** | *22q11DS* | 36 | **.44** | **.007** | 0.13 – 0.67 | 36 | .01 | .96 | 0.32 – 0.34 | 35 | .07 | .68 | -0.27 – 0.40 |
|  | *TD* | 78 | **.63** | **.001** | 0.47 – 0.74 | 77 | .05 | .67 | -0.18 – 0.27 | 78 | .12 | .30 | -0.11 – 0.33 |
| **WM BW** | *22q11DS* | 36 | .32 | .058 | -0.11 – 0.59 | 36 | -.07 | .70 | -0.39 – 0.27 | 35 | .31 | .067 | -0.02 – 0.59 |
|  | *TD* | 77 | **.63** | **<.001** | 0.47 – 0.75 | 76 | .16 | .17 | -0.07 – 0.37 | 77 | .17 | .14 | -0.06 – 0.38 |

Abbreviations: BW = Backward, CI = Confidence Interval, FW = Forward, IQ = Intelligence Quotient, SA = Selective Attention, SES = Socioeconomic status, TD = Typically Developing, WM = Working Memory.
Note. Pearson bivariate correlations, significant correlations are in bold.
* = This is Spearman’s Rho as these non-parametric outcomes differed from the Pearson correlation (*r*(42) = .35, *p* =.023, CI 95% [0.05 – 0.59])).

**Appendix D – SA outcomes per display**

**Table 9.** Results of the SA task for children with 22q11DS (*n* = 42) and TD children (*n* = 81) per display.

|  |  | **Hits** | | | **Errors** | | | **Repetitions** | | |
| --- | --- | --- | --- | --- | --- | --- | --- | --- | --- | --- |
|  |  | *M* | *SD* | *range* | *M* | *SD* | *range* | *M* | *SD* | *range* |
| **Display 1** | 22q11DS | 6.10 | 1.59 | 2-8 | 0.55 | 0.83 | 0-3 | 0.17 | 0.58 | 0-3 |
|  | TD | 6.83 | 1.4 | 2-8 | 0.21 | 0.49 | 0-2 | 0.07 | 0.31 | 0-2 |
|  |  |  |  |  |  |  |  |  |  |  |
| **Display 2** | 22q11DS | 6.50 | 1.31 | 4-8 | 0.36 | 0.58 | 0-2 | 0.10 | 0.48 | 0-3 |
|  | TD | 7.12 | 1.02 | 5-8 | 0.16 | 0.43 | 0-2 | 0.12 | 0.33 | 0-1 |
|  |  |  |  |  |  |  |  |  |  |  |
| **Display 3** | 22q11DS | 5.33 | 1.66 | 1-8 | 0.52 | 0.89 | 0-4 | 0.14 | 0.35 | 0-1 |
|  | TD | 6.25 | 1,47 | 3-8 | 0.11 | 0.35 | 0-2 | 0.10 | 0.34 | 0-2 |
|  |  |  |  |  |  |  |  |  |  |  |
| **Display 4** | 22q11DS | 2.02 | 1.26 | 0-5 | 0.45 | 0.74 | 0-3 | 0.07 | 0.26 | 0-1 |
|  | TD | 2.53 | 1.41 | 0-7 | 0.09 | 0.32 | 0-2 | 0.01 | 0.11 | 0-1 |

Abbreviations: TD = Typically Developing.
Note. For display 1-3 the maximum number of Hits is 8, for display 4 max. Hits is 9, and the max. is 33 for the total number of Hits. There was no maximum to the number of Errors and Repetitions.

**Appendix E – HTKS task completion comparison**

**Table 10.** Comparison of demographic variables between children with and without complete HTKS data for both groups.

|  |  | **HTKS complete** | | **HTKS missing** | |  |
| --- | --- | --- | --- | --- | --- | --- |
|  | *22q11DS* | *n* = 9 | | *n* = 35 | |  |
|  | *TD* | *n* = 58 | | *n* = 23 | |  |
|  |  | *M* | *SD* | *M* | *SD* |  |
| **Age** | *22q11DS* | 5.8 | 0.3 | 4.7 | 1.0 | *t*(39.060) = -5.49, *p* < .001, *g* = 1.21, 95% CI [-1.55 – -0.72] |
|  | *TD* | 5.0 | 0.7 | 3.7 | 0.6 | *t*(45.004) = -8.02, *p* < .001, *g* = 1.93, 95% CI [-1.63 – -0.98] |
| **Sex** | *22q11DS* | *n* f/m = 5/4 | | *n* f/m = 14/21 | | *χ^2^*(1) = .71, *p* = .40, *V* = .13 |
|  | *TD* | *n* f/m = 32/26 | | *n* f/m = 13/10 | | *χ^2^*(1) = .01, *p* = .91, *V* = .01 |
| **IQ** | *22q11DS* | 82.3 | 11.4 | 79.7 | 11.9 | *t*(13.128) = -.62, *p* = .55, *g* = .22, 95% CI [-12.02 – 6.69] |
|  | *TD* | 107.5 | 12.1 | 100.8 | 15.7 | *t*(30.848) = -1.81, *p* = .081, *g* = .51, 95% CI [-14.28 – 0.86]* |
| **SES** | *22q11DS* | 6.4 | 1.5 | 6.4 | 1.9 | *t*(15.312) = -0.00, *p* = 1.0, *g* = .0, 95% CI [-1.24 – 1.23] |
|  | *TD* | 8.0 | 1.2 | 7.5 | 1.5 | *t*(34.237) = -1.34, *p* = .19, *g* = .39, 95% CI [-1.17 – 0.24] |

Abbreviations: IQ = Intelligence Quotient, SD = Standard Deviation, SES = Socioeconomic status, TD = Typically Developing

* Different from parametric: *t*(78) = -2.035, *p* = .045, *d* = .51, 95% CI [-13.27 – -0.15] and Mann-Whitney U (*U* = 400.5, *p* = .010).

**Appendix F – Task completion CHD group comparison**

**Table 11.** Comparison of task completion and demographic variables between children with 22q11DS with and without HS-CHD

|  | **CHD** | | **No HS-CHD** | |  |
| --- | --- | --- | --- | --- | --- |
| **Task completion** | Not complete | Complete | Not complete | Complete |  |
|  | 3 | 13 | 5 | 23 | *χ^2^*(1) = .01, *p* = .94, *V* = .01 |
|  |  |  |  |  |  |
| **Sex** | *F* | *M* | *F* | *M* |  |
|  | 6 | 10 | 13 | 15 | *χ2*(1) = .33, *p* = .57, *V* = .09 |
|  |  |  |  |  |  |
|  | *M* | *SD* | *M* | *SD* |  |
| **Age** | 4.96 | 1.06 | 4.86 | 1.04 | *t*(30.759) = .10, *p* = .76, *g* = .10, 95% CI [-0.77 – 0.57] |
| **SES** | 6.75 | 1.33 | 6.27 | 1.97 | *t*(40.713) = .93, *p* = .34, *g* = .27, 95% CI [-1.49 – 0.53] |
| **IQ** | 75.4 | 12.2 | 82.9 | 10.7 | *t*(25.963) = 4.00, *p* = .056, *g* = .67, 95% CI [-0.21 – 15.27] * |

Abbreviations: HS-CHD = Hemodynamically Significant Congenital Heart Defect, IQ = Intelligence Quotient, SD = Standard Deviation, SES = Socioeconomic status, TD = Typically Developing

* Different from parametric: *t*(40) = 2.08, *p* = .044, *d* = .65, 95% CI [0.20 – 14.85] and Mann-Whitney U (*U* = 111.5, *p* = .017).

**Appendix G – Sensitivity analyses CHD comparison**

In the primary analysis, we used the contrast presence of hemodynamically significant CHD (HS-CHD) versus absence of HS-CHD (being the sum of hemodynamically insignificant CHD and no CHD). These sensitivity analyses used different grouping criteria for the CHD classification in the children with 22q11DS. In the first analysis, children with any type of cardiac anomaly (CA) were compared to children without cardiac anomalies. In the second analysis, children who had undergone cardiac surgery were compared to children who had not undergone surgery for cardiac corrections. There were two cases of children with aberrant subclavian arteries that were surgically corrected due esophageal compression, but who did not have hemodynamically significant CHD. See table 12 for EF outcomes for the respective groups.

*Sensitivity analysis 1: Group comparisons between children with 22q11DS with and without any Cardiac Anomaly (CA)*There was no effect of *CA* on the SA task (*V* = 0.17, *F*(3, 38) = 2.57, *p* = .069, *η_p_^2^* = .17). These findings change when *Age*, *SES*, and *IQ* were entered as covariates (*V* = 0.21, *F*(3, 34) = 3.07, *p* = .041, *η_p_^2^* = .21), although none of the covariates were significant. In the model with covariates, children with *CA* had more *Errors* (*F*(1, 40) = 4.49, *p* = .041, *η_p_^2^* = .11) than children without any *CA* (see Table 2). There was no difference on the total number of *Hits* (*F*(1, 40) = 1.60, *p* = .21, *η_p_^2^* = .043) and *Repetitions* between the groups (*F*(1, 40) = .61, *p* = .44, *η_p_^2^* = .02). Results should be interpreted with caution as the assumption of homogeneity of covariance matrices was violated.
 There was no effect of *CA* on the WM task (*V* = 0.08, *F*(2, 33) = 1.37, *p* = .27, *η_p_^2^* = .08). These findings did not change when *Age*, *SES*, and *IQ* were entered as covariates. *Age* was a significant covariate (*V* = 0.31, *F*(2, 29) = 6.56, *p* = .004, *η_p_^2^* = .31) but did not change the effect of *CA*.

*Sensitivity analysis 2: Group comparisons between children with 22q11DS with and without cardiac surgery*There was no effect of *Cardiac Surgery* on the SA task (*V* = 0.13, *F*(3, 38) = 1.90, *p* = .15, *η_p_^2^* = .13). These findings did not change when *Age*, *SES*, and *IQ* were entered as a covariate, nor were they significant covariates. Results should be interpreted with caution as the assumption of homogeneity of covariance matrices was violated.
 There was no effect of *Cardiac Surgery* on the WM task (*V* = 0.02, *F*(2, 33) = .36, *p* = .70, *η_p_^2^* = .02). These findings did not change when *Age*, *SES*, and *IQ* were entered as covariates. *Age* was a significant covariate (*V* = 0.30, *F*(2, 29) = 6.06, *p* = .006, *η_p_^2^* = .30) but did not change the effect of *Cardiac Surgery*.

**Table 12.** EF results of children with 22q11DS with and without cardiac surgery

|  |  | **CA** | **No CA** |  | **Surgery** | **No surgery** |
| --- | --- | --- | --- | --- | --- | --- |
| **SA Hits** | *N* | 24 | 18 |  | 17 | 25 |
|  | *M* | 20.54 | 19.17 |  | 20.06 | 19.88 |
|  | *SD* | 4.05 | 4.91 |  | 4.31 | 4.61 |
| **SA Errors** | *N* | 24 | 18 |  | 17 | 25 |
|  | *M* | 2.38 | 1.22 |  | 2.53 | 1.44 |
|  | *SD* | 2.34 | 1.52 |  | 2.40 | 1.76 |
| **SA Repetitions** | *N* | 24 | 18 |  | 17 | 25 |
|  | *M* | 0.67 | 0.22 |  | 0.82 | 0.24 |
|  | *SD* | 1.27 | 0.55 |  | 1.47 | 0.52 |
|  |  |  |  |  |  |  |
| **WM Forward** | *N* | 22 | 14 |  | 15 | 21 |
|  | *M* | 2.68 | 3.14 |  | 2.8 | 2.9 |
|  | *SD* | 0.78 | 0.86 |  | 0.86 | 0.83 |
| **WM Backward** | *N* | 22 | 14 |  | 15 | 21 |
|  | *M* | 1.73 | 1.93 |  | 1.67 | 1.9 |
|  | *SD* | 0.70 | 1.0 |  | 0.72 | 0.89 |

Abbreviations: CA = Cardiovascular Anomaly, SA = Selective Attention, WM = Working Memory.
Note. The maximum of SA Hits is 33, that of WM Forward is 9, and that of WM Backward is 6. SA Errors and SA Repetitions have no maximum.
